# Conversion therapy followed by curative resection in a patient with ACTH-secreting pancreatic neuroendocrine tumor G3: a case report

**DOI:** 10.3389/fmed.2026.1875610

**Published:** 2026-09-22

**Authors:** Bo Zhou, Fei Xiang, Sheng Yan

**Affiliations:** Department of Hepatobiliary and Pancreatic Surgery, The Second Affiliated Hospital, Zhejiang University School of Medicine, Hangzhou, China

**Keywords:** case report, complete surgical removal, conversion therapy, ectopic ACTH secretion, pancreatic ueuroendocrine tumor

## Abstract

**Background:**

Pancreatic neuroendocrine tumors (pNETs) with ectopic adrenocorticotropic hormone (ACTH) production are uncommon, highly aggressive, and carry a dismal prognosis. Early nonspecific symptoms often delay diagnosis, and treatment strategies remain controversial. For patients with locally advanced pNETs, conversion therapy has only rarely been reported and remains a sparingly applied strategy.

**Case presentation:**

A 37-year-old woman presented with a six-month history of progressive swelling in both lower extremities and her face. Further workup confirmed ACTH-dependent Cushing syndrome. CT and MRI identified a 5.7 × 2.8 cm lesion in the pancreatic body/tail that invaded the left adrenal gland and splenic vessels, accompanied by retroperitoneal lymph node spread. Biopsy results indicated a G3 pNET. Given the borderline resectable status due to local advancement, she received conversion therapy comprising a long-acting somatostatin analogue followed by CapTem chemotherapy. After three months of neoadjuvant treatment, imaging showed modest primary tumor shrinkage and marked reduction of lymph node size, reflecting successful downstaging. Subsequently, she underwent distal pancreatectomy with splenectomy, left adrenalectomy, and lymph node dissection, achieving R0 resection. Final pathology confirmed a G3 pNET (Ki-67 30%–40%) with positive SSTR2 and ACTH immunostaining. Although the patient eventually experienced disease progression and succumbed to COVID-19 infection 37 months after initial diagnosis, this case illustrates that conversion therapy can render an initially borderline resectable high-grade pNET amenable to curative resection.

**Conclusion:**

This report underscores the aggressive trajectory of ACTH-secreting pNET G3 and suggests the potential utility of conversion therapy. Further studies are needed to validate this approach.

## Introduction

Pancreatic neuroendocrine tumors (pNETs) originate from pancreatic islet cells and constitute roughly 1%–3% of all pancreatic neoplasms (1, 2). While many pNETs are non-functional, a fraction secrete bioactive hormones such as insulin, gastrin, glucagon, or adrenocorticotropic hormone (ACTH). Ectopic ACTH secretion by pNETs is an exceptionally rare driver of ACTH-dependent Cushing syndrome, accounting for fewer than 1.2% of all pNETs in Japanese cohorts and only 4%–16% of ectopic ACTH syndrome cases overall (3–6).

ACTH-producing pNETs behave aggressively, with the majority of patients already harboring metastases at initial presentation. Their 5-year survival is poor, ranging from 16% to 35%, which is substantially worse than that of other functioning pNETs (3, 5). Diagnosis requires biochemical proof of ACTH-dependent hypercortisolism followed by imaging to pinpoint the pancreatic tumor, typically employing computed tomography, magnetic resonance imaging, and somatostatin receptor-based modalities such as 68Ga-DOTATATE PET/CT (7).

Management involves two parallel goals: controlling life-threatening hypercortisolemia (often using steroidogenesis inhibitors like metyrapone or ketoconazole) and treating the underlying neuroendocrine neoplasm. Somatostatin analogues, systemic chemotherapy (e.g., capecitabine plus temozolomide), targeted agents (everolimus, sunitinib), and peptide receptor radionuclide therapy (PRRT) have been employed with varying degrees of success (8–11). For patients with locally advanced pNETs, conversion therapy has only rarely been reported and remains a sparingly applied strategy. In selected cases, it may reduce tumour burden, achieve downstaging, and enable subsequent curative resection, but clinical experience and evidence remain extremely limited. Here we describe a case of an initially borderline resectable ACTH-secreting pNET G3 that was successfully downstaged with a somatostatin analogue and CapTem chemotherapy, followed by R0 resection, suggests the potential utility of conversion therapy.

## Case presentation

A 37-year-old woman presented with six months of progressive edema involving both lower extremities and her face. She had no significant past medical history. On admission, physical examination was unremarkable apart from mild edema, and blood pressure was normal. Initial laboratory studies showed normal tumor markers, hypokalemia (2.69 mmol/L), and hyperglycemia (8.86 mmol/L). Additional abnormalities included anemia (hemoglobin 83 g/L), hypoalbuminemia (albumin 33.1 g/L), and elevated total cholesterol (6.36 mmol/L). An endocrine workup, prompted by persistent electrolyte and glucose disturbances, revealed markedly elevated serum cortisol and plasma ACTH levels without diurnal variation (Table 1). Thyroid function tests indicated central hypothyroidism (low TT3, FT3, TT4, FT4 with normal TSH). Sex hormone panel was normal, and pituitary MRI showed no significant abnormalities. Fasting C-peptide was low (0.12 nmol/L), and glycated hemoglobin (HbA1c) was elevated (7.5%), consistent with newly diagnosed diabetes mellitus.

**Table 1:** Endocrine hormone test results.

Item	Value	Reference range
Cortisol (0am)	652.9 nmol/L	-
Cortisol (8am)	813.9 nmol/L	154.0–638.0 nmol/L
Cortisol (4pm)	750.0 nmol/L	79–388 nmol/L
Adrenocorticotropic hormone (0am)	241.2 nmol/L	-
Adrenocorticotropic hormone (8am)	268.5 nmol/L	7.2–63.3 nmol/L
Adrenocorticotropic hormone (4pm)	284.7 nmol/L	-
24-hour urinary potassium	181.97 mmol/24h	25–100 mmol/24h
24-hour urinary free cortisol	1,499.1 μg/24h	19.30–317.50 μg/24h
Total triiodothyronine (TT3)	0.48 nmol/L	0.89–2.44 nmol/L
Free T3 (FT3)	<1.54 pmol/L	2.63–5.70 pmol/L
Total thyroxine (TT4)	46.3 nmol/L	58.1–140.6 nmol/L
Free thyroxine (FT4)	8.62 pmol/L	9.01–19.05 pmol/L
High-sensitivity thyroid-stimulating hormone (TSH)	0.47 mIU/L	0.35–4.94 mIU/L

Contrast-enhanced abdominal CT demonstrated a large 5.7 × 2.8 cm mass in the pancreatic body/tail, invading the left adrenal gland and splenic vessels. Retroperitoneal lymph node metastases adjacent to the left renal vessels and abdominal aorta were present, along with left-sided regional portal hypertension, gastric varices, and splenomegaly (Figures 1A,B). MRI confirmed these findings (Figures 2A,C). Endoscopic ultrasound-guided biopsy of the pancreatic lesion showed a few atypical epithelioid cells. Immunohistochemical staining revealed diffuse positivity for synaptophysin, chromogranin, CD56, ATRX, and CK(AE1/AE3), with negative reactions for Vimentin and p53. The Ki-67 proliferative index was 20%–30% in hot spots, with a mitotic count of 1.7 per 2 mm^2^. Somatostatin receptor type 2 (SSTR2) was also positive. The tumor was classified as pNET G3 with concurrent ectopic Cushing syndrome.

**Figure 1:**
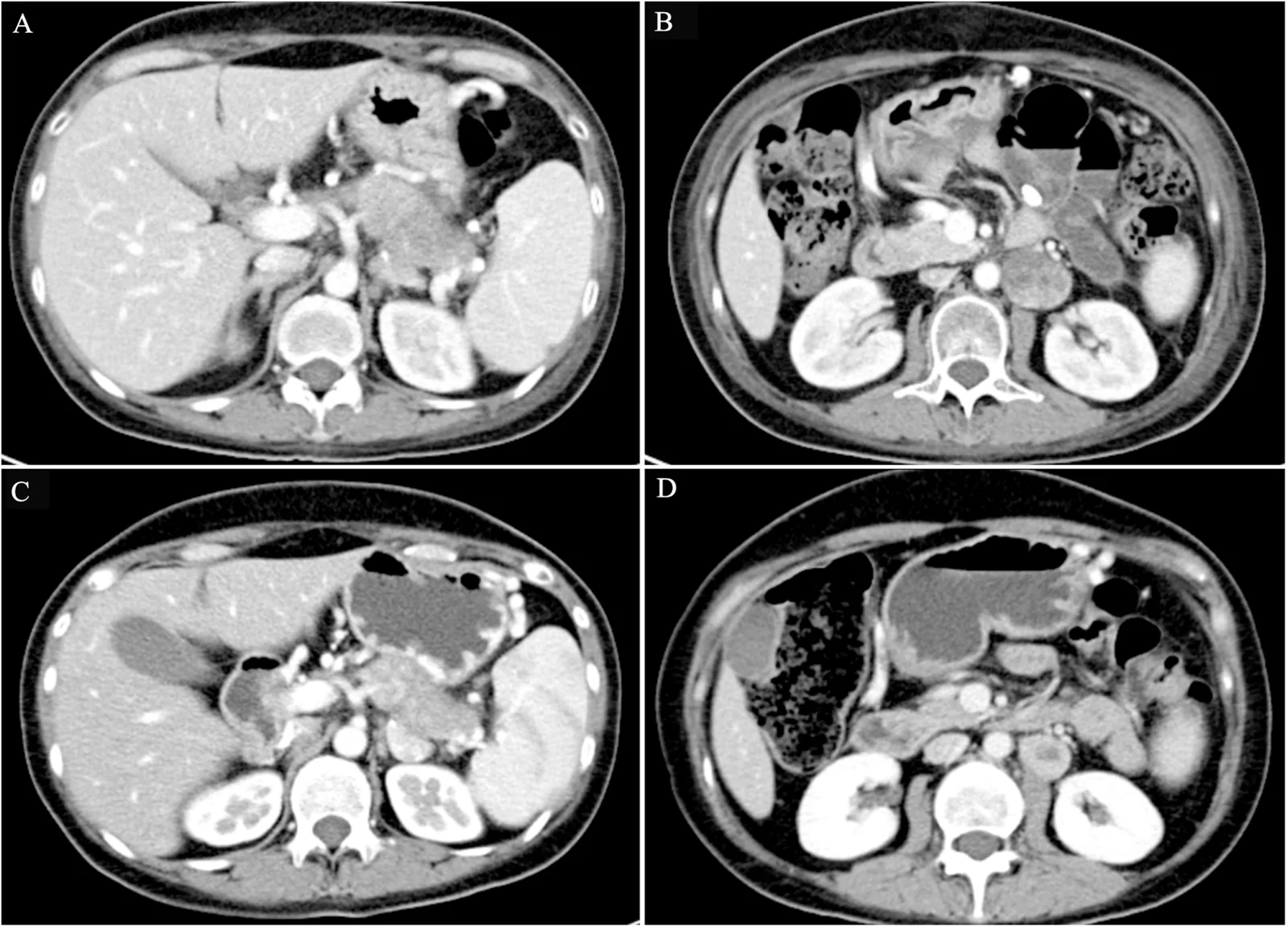
Abdominal contrast-enhanced CT at diagnosis **(A,B)** showing a 5.7 × 2.8 cm pancreatic body/tail mass invading the left adrenal and splenic vessels, with retroperitoneal lymphadenopathy, left-sided portal hypertension, gastric varices, and splenomegaly. After 3 months of neoadjuvant therapy **(C,D)**, the primary lesion shrank mildly, and lymph node size markedly decreased.

**Figure 2:**
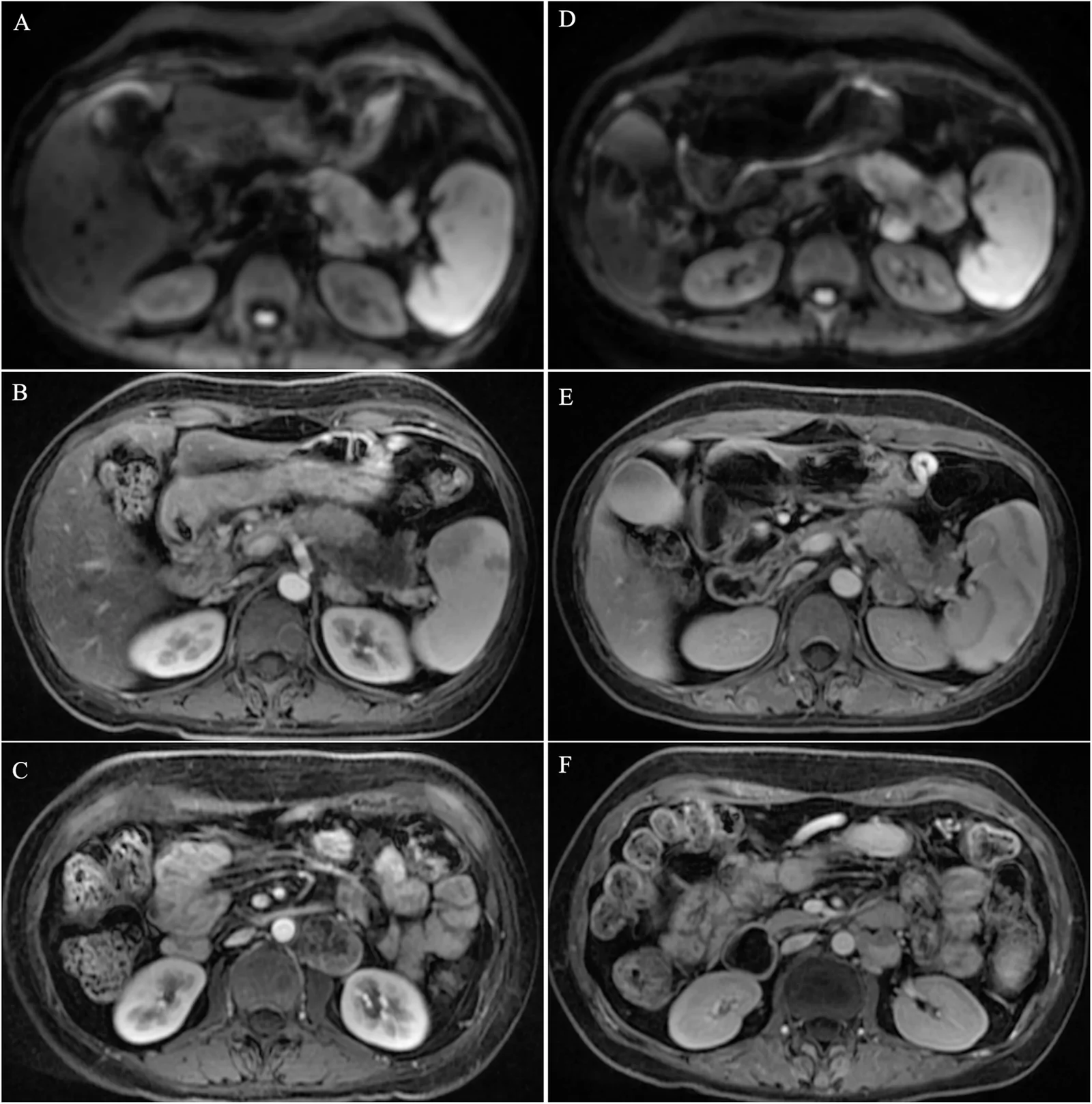
Abdominal MRI before treatment **(A–C)** confirmed the pancreatic mass with invasion of the left adrenal and splenic vessels, along with multiple retroperitoneal nodal metastases. Following 3 months of neoadjuvant therapy **(D–F)**, the tumor showed modest size reduction, and the lymph nodes regressed significantly.

### Treatment and follow-up

The tumor was deemed borderline resectable due to splenic vessel abutment (no encasement), adrenal invasion, and extensive retroperitoneal lymphadenopathy. Although no standardized definition exists for locally advanced pNETs, R0 resection would require multivisceral resection, and upfront surgery carried high morbidity risk given uncontrolled hypercortisolism. Thus, a conversion therapy strategy was adopted. Although steroidogenesis inhibitors such as metyrapone or ketoconazole are commonly used for hypercortisolism control in ectopic ACTH syndrome, we chose somatostatin analogue as first-line therapy due to the positive SSTR2 staining on biopsy. Within two weeks, her serum potassium level rose from 2.69 mmol/L to 3.80 mmol/L, and fasting blood glucose decreased from 8.86 mmol/L to 6.20 mmol/L. She then received CapTem chemotherapy (capecitabine 1.0 g twice daily on days 1–14 and temozolomide 280 mg once daily on days 10–14, every 28 days). After two cycles of CapTem (approximately 8 weeks from the start of treatment), her serum cortisol level decreased from 813.9 nmol/L to 154.7 nmol/L, plasma ACTH decreased from 268.5 nmol/L to 49.8 nmol/L, and 24-hour urinary free cortisol dropped from 1,499.1 μg/24 h to 102.3 μg/24 h, all within or near the normal reference ranges. After three months of neoadjuvant therapy, repeat CT (Figures 1C,D) and MRI (Figures 2D,F) showed mild shrinkage of the primary tumor and a marked decrease in the size of the retroperitoneal lymph nodes, indicating successful downstaging.

Following multidisciplinary discussion, she underwent distal pancreatectomy with splenectomy, left adrenalectomy, and retroperitoneal lymph node dissection. R0 resection was achieved with negative margins. Histologically, the tumor demonstrated a trabecular and nested growth pattern with monomorphic epithelioid cells exhibiting finely granular chromatin and moderate eosinophilic cytoplasm, consistent with a well-differentiated morphology. No evidence of small cell or large cell features, geographic necrosis, or high-grade nuclear atypia was identified, which helped distinguish this tumor from poorly differentiated neuroendocrine carcinoma (NEC). The mitotic count was 35 per 2 mm^2^, and the Ki-67 index was 30%–40% in hot spots. Perineural and multifocal vascular invasion were present. The tumor invaded the adrenal gland but not the spleen. Six tumor deposits were found around the pancreas. Immunohistochemistry showed diffuse positivity for synaptophysin, chromogranin, ACTH, SSTR2, CD56, ATRX, and CK(AE1/AE3), with negative reactions for Vimentin and p53 (Figure 3). Finally, the diagnosis of pNET grade 3 (G3) with ectopic Cushing syndrome was made according to the WHO 2019 classification. Postoperatively, the long-acting somatostatin analogue was continued, but CapTem was discontinued after two cycles due to intolerance.

**Figure 3:**
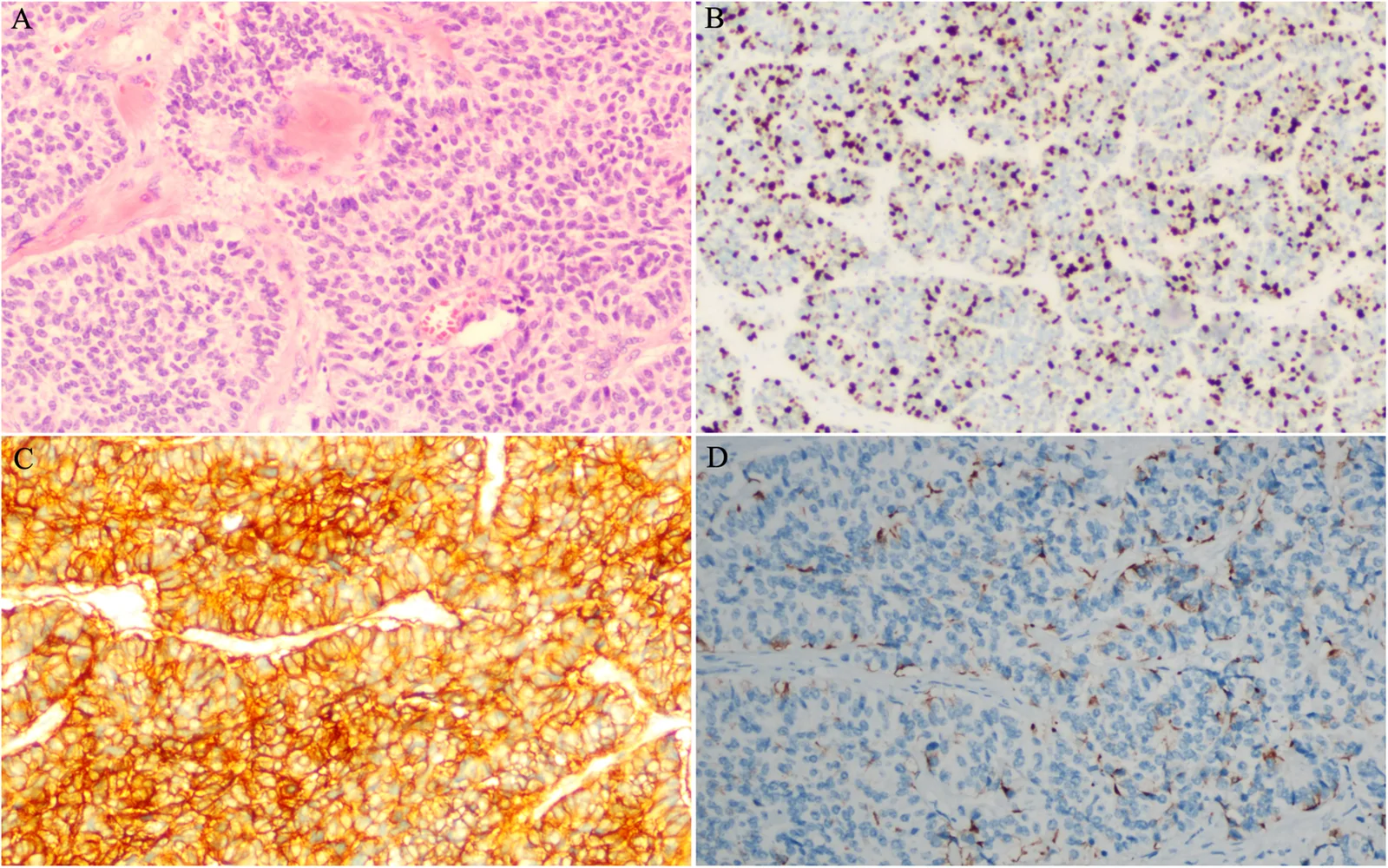
Postoperative histopathology (**A**, H&E, ×200) demonstrated a well-differentiated tumor composed of atypical epithelioid cells. The Ki-67 labeling index **(B)** was 30−40% in hot spots. Immunohistochemistry revealed positivity for SSTR2 **(C)** and ACTH (**D**, ×200), confirming an ACTH-secreting pNET.

Ten months after surgery, CT identified a new 1.8 cm left hilar lymph node that had enlarged compared with prior imaging, along with multiple small retroperitoneal and mesenteric nodes. Radiotherapy was delivered to the left hilar node, and the somatostatin analogue was continued. The irradiated nodule shrank slightly. However, 12 months later, upper abdominal MRI detected a new right adrenal nodule suspicious for metastasis, as well as multiple enlarged nodes in the porta hepatis, retroperitoneum, mesentery, and peripancreatic region. A somatostatin receptor PET-CT with ^1^⁸F-octreotide confirmed increased uptake in the right adrenal, liver, left adnexa, and multiple lymph nodes, consistent with widespread metastatic disease (Figure 4).

**Figure 4:**
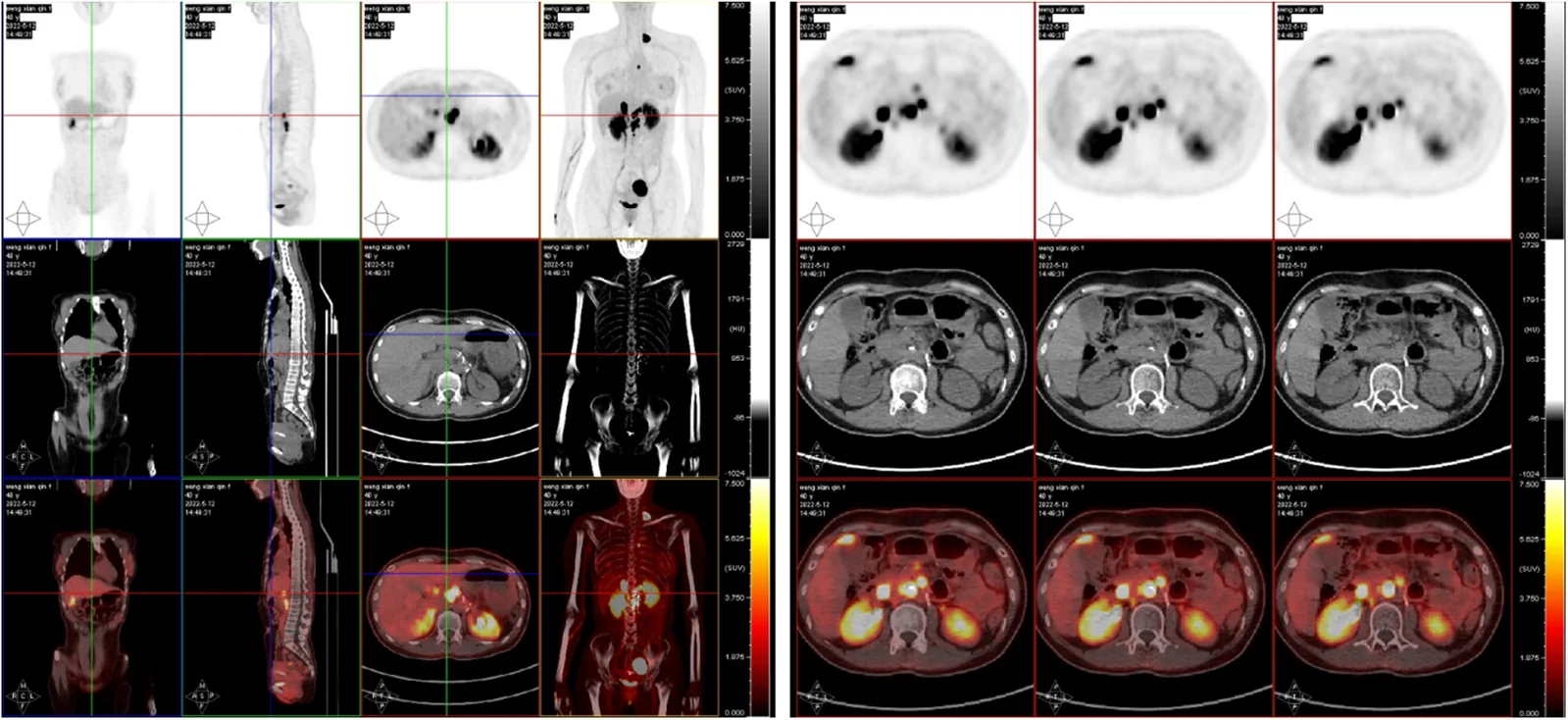
^1^⁸F-octreotide somatostatin receptor PET-CT showed tracer uptake in the right adrenal, liver, left adnexa, and multiple lymph nodes, indicative of widespread metastatic disease.

Treatment was then switched to capecitabine plus surufatinib 300 mg once daily, together with the long-acting somatostatin analogue. Initial follow-up MRI showed reduction of the right adrenal nodule and stable lymph nodes. However, CT performed 8 months later demonstrated progression of the retroperitoneal lymph nodes, which had increased in both size and number. The patient was advised to consider peptide receptor radionuclide therapy (PRRT). Unfortunately, she ultimately died from COVID-19 infection 37 months after her initial diagnosis.

## Discussion

Ectopic ACTH-producing pNETs are known for their aggressive clinical course, with most patients already exhibiting metastases at the time of diagnosis. We describe a 37-year-old woman with an ACTH-secreting pNET grade 3 (G3) and retroperitoneal lymph node metastases, whose tumor was curatively resected after successful downstaging.

According to the WHO 2019 classification, pNET G3 is defined by a Ki-67 index >20% and/or mitotic count >20 per 2 mm^2^, with retained well-differentiated morphology. This distinction from poorly differentiated NEC is clinically important, as G3 NETs and NECs respond differently to chemotherapy and targeted agents. In our case, trabecular/nested architecture, monomorphic cells with fine chromatin, a Ki-67 of 30-40%, and a mitotic count of 35 per 2 mm^2^ confirmed well-differentiated G3 NET. Retained ATRX and absent p53 further supported this classification (3, 12). Morphologically, well-differentiated NETs show organoid patterns, monomorphic cells, fine chromatin, and little necrosis; NECs exhibit sheet-like growth, high atypia, prominent nucleoli, and abundant necrosis. Immunohistochemistry aids discrimination: retained ATRX and absent p53 favor NET, though these markers are not entirely specific. This distinction has direct therapeutic implications: well-differentiated G3 NETs are more likely to respond to temozolomide-based regimens, whereas NECs are treated with platinum/etoposide. Misclassification may lead to suboptimal therapy.

It has been reported that patients with ectopic ACTH syndrome often lack the classic physical hallmarks of Cushing's syndrome, such as moon face, buffalo hump, or abdominal striae, particularly when the disease follows a rapidly progressive course (6). In our patient, the initial symptoms of bilateral lower-limb and facial edema were nonspecific and could easily have been attributed to other causes. Nevertheless, the persistence of refractory hypokalemia together with hyperglycemia eventually triggered a comprehensive endocrine evaluation that uncovered pronounced ACTH-dependent hypercortisolism. The absence of typical cushingoid features at the outset likely contributed to diagnostic delay, a well-recognized challenge in this condition (7). In patients with severe hypercortisolism, prompt medical control is essential prior to any surgical intervention. Steroidogenesis inhibitors such as metyrapone (which inhibits 11β-hydroxylase) and ketoconazole (which inhibits multiple cytochrome P450 enzymes involved in cortisol synthesis) are the mainstay of medical therapy. Metyrapone is particularly useful for rapid cortisol reduction, while ketoconazole is often used in combination therapy for refractory cases. In our case, we opted for somatostatin analogue therapy due to the positive SSTR2 staining on biopsy, which not only controlled ACTH secretion but also allowed us to avoid the potential toxicities of steroidogenesis inhibitors.

Given the absence of established criteria for borderline resectability in pNETs, our decision was based on anatomical factors (vascular involvement and adrenal invasion) and clinical factors (severe hypercortisolism), as previously suggested by case series on locally advanced functioning pNETs (3). This case highlights the potential role of conversion therapy in managing locally advanced, high-grade ACTH-secreting pNETs. Our patient initially presented with borderline resectable disease due to adjacent structure invasion and regional lymph node metastases. After three months of somatostatin analogue and CapTem chemotherapy, biochemical normalization and a partial radiographic response (marked nodal regression and modest tumor shrinkage) enabled subsequent R0 resection. Platinum/etoposide remains standard for poorly differentiated NEC, whereas well-differentiated G3 NETs respond better to temozolomide-based regimens (e.g., CapTem), with reported response rates of 40%–70%. FOLFIRINOX lacks robust evidence and is generally reserved for salvage. In our patient, well-differentiated morphology, retained ATRX, and absent p53 supported CapTem over platinum, consistent with the observed radiographic response. Neoadjuvant CapTem in pNET G3 is rarely documented. Strosberg reported a 70% response in metastatic disease (13), but experience in borderline resectable settings is limited to case reports, including one by Zhang (8) describing biochemical remission after neoadjuvant SSA plus chemotherapy. Our case adds to this sparse evidence by demonstrating dual biochemical and radiographic downstaging with SSA+CapTem in a high-grade functioning pNET. Despite initial downstaging, new contralateral adrenal and liver metastases appeared 22 months after resection—a pattern consistent with the aggressive biology of ACTH-secreting pNETs (4, 10). Subsequent addition of surufatinib plus capecitabine provided transient disease control before progression (12, 13), but its efficacy in high-grade, ACTH-secreting pNETs remains unclear.

Several pathological features in our case predicted recurrence: a high Ki-67 index (30-40%), a well-established risk factor in pNETs when exceeding 10%; perineural and multifocal vascular invasion, associated with increased metastatic potential; six tumor deposits around the pancreas, indicating aggressive locoregional spread; and Ki-67 discordance between biopsy (20-30%) and surgical specimen (30-40%), suggesting intratumoral heterogeneity and possible tumor progression during neoadjuvant therapy. Collectively, these placed the patient in a high-risk category, consistent with Maragliano (3), who reported a 5-year survival of only 20% for ACTH-secreting pNETs with G3 grade and vascular invasion, versus 60% for G1/G2 tumors. The early recurrence at 10 months reflects the aggressive nature of this entity and argues for close surveillance and adjuvant consideration in similar cases. Although postoperative somatostatin analogue was administered, the recurrence pattern suggests that more intensive systemic therapy or early PRRT may be warranted in high-risk patients.

Reports of successful conversion therapy in ACTH-secreting pNET G3 are extremely scarce (14). Unlike most published cases, which present with unresectable metastases at diagnosis (3, 4), our patient had locally advanced but potentially downstageable disease. This enabled curative-intent resection after neoadjuvant therapy, contributing to a 37-month overall survival—favorable compared with the reported median of 19-24 months for metastatic ACTH-secreting pNET G3. While a single case cannot establish efficacy, our experience suggests that conversion therapy merits consideration in selected patients. Decisions should be individualized by a multidisciplinary team, weighing patient condition, tumor biology, and institutional expertise. Prospective studies are needed to define optimal selection criteria and regimens. It is worth noting that hypercortisolism and chemotherapy induces significant immunosuppression, increasing vulnerability to severe infectious complications (15). In the context of a global pandemic, her immunocompromised status likely contributed to a fatal outcome from a viral illness. Future studies should explore optimal neoadjuvant regimens and patient selection criteria for conversion therapy in this rare tumor entity.

## Conclusion

ACTH-secreting pNET G3 is a rare and highly aggressive malignancy associated with a poor prognosis. Early recognition of subtle hypercortisolism manifestations, timely biochemical confirmation, and a multidisciplinary treatment approach are essential. For patients with locally advanced, borderline resectable disease, conversion therapy using somatostatin analogues and CapTem chemotherapy may achieve tumor downstaging and enable curative resection, offering a window of disease control. Although most patients eventually experience progression, the ability to achieve R0 resection after neoadjuvant treatment may improve survival compared with palliative approaches.

## Data Availability

The raw data supporting the conclusions of this article will be made available by the authors, without undue reservation.

## References

[1] Ma Z-Y Gong Y-F Zhuang H-K Zhou Z-X Huang S-Z Zou Y-P. Pancreatic neuroendocrine tumors: a review of serum biomarkers, staging, and management. World J Gastroenterol. (2020) 26:2305–22. 10.3748/wjg.v26.i19.2305 32476795 PMC7243647

[2] Konukiewitz B Jesinghaus M Kasajima A Klöppel G. Neuroendocrine neoplasms of the pancreas: diagnosis and pitfalls. Virchows Arch. (2022) 480:247–57. 10.1007/s00428-021-03211-5 34647171 PMC8986719

[3] Maragliano R Vanoli A Albarello L Milione M Basturk O Klimstra DS. ACTH-secreting pancreatic neoplasms associated with cushing syndrome: clinicopathologic study of 11 cases and review of the literature. Am J Surg Pathol. (2015) 39:374–82. 10.1097/PAS.0000000000000340 25353285

[4] Patel FB Khagi S Daly KP Lechan RM Ummaritchot V Saif MW. Pancreatic neuroendocrine tumor with ectopic adrenocorticotropin production: a case report and review of literature. Anticancer Res. (2013) 33:4001–5. 24023341

[5] Clark ES Carney JA. Pancreatic islet cell tumor associated with cushing’s syndrome. Am J Surg Pathol. (1984) 8:917–24. 10.1097/00000478-198412000-00004 6097131

[6] Ilias I Torpy DJ Pacak K Mullen N Wesley RA Nieman LK. Cushing’s syndrome due to ectopic corticotropin secretion: twenty years’ experience at the national institutes of health. J Clin Endocrinol Metab. (2005) 90:4955–62. 10.1210/jc.2004-2527 15914534

[7] Isidori AM Sbardella E Zatelli MC Boschetti M Vitale G Colao A. Conventional and nuclear medicine imaging in ectopic cushing’s syndrome: a systematic review. J Clin Endocrinol Metab. (2015) 100:3231–44. 10.1210/JC.2015-1589 26158607 PMC4570166

[8] Zhang C Jin J Xie J Ye L Su T Jiang L. The clinical features and molecular mechanisms of ACTH-secreting pancreatic neuroendocrine tumors. J Clin Endocrinol Metab. (2020) 105:dgaa507. 10.1210/clinem/dgaa507 32785672

[9] Kondo T Matsuyama R Ashihara H Matsuo Y Sasaki K Goto R. A case of ectopic adrenocorticotropic hormone-producing pancreatic neuroendocrine tumor with multiple liver metastases. Endocr J. (2010) 57:229–36. 10.1507/endocrj.k09e-179 20032567

[10] Rajeev SP McDougall S Terlizzo M Palmer D Daousi C Cuthbertson DJ. Evolution in functionality of a metastatic pancreatic neuroendocrine tumour (pNET) causing cushing’s syndrome: treatment response with chemotherapy. BMC Endocr Disord. (2014) 14:70. 10.1186/1472-6823-14-70 25151270 PMC4149042

[11] Kashyap R Hofman MS Michael M Kong G Akhurst T Eu P. Favourable outcomes of (177)Lu-octreotate peptide receptor chemoradionuclide therapy in patients with FDG-avid neuroendocrine tumours. Eur J Nucl Med Mol Imaging. (2015) 42:176–85. 10.1007/s00259-014-2906-4 25209134

[12] Basturk O Yang Z Tang LH Hruban RH Adsay V McCall CM. The high-grade (WHO G3) pancreatic neuroendocrine tumor category is morphologically and biologically heterogenous and includes both well differentiated and poorly differentiated neoplasms. Am J Surg Pathol. (2015) 39:683–90. 10.1097/PAS.0000000000000408 25723112 PMC4398606

[13] Strosberg JR Fine RL Choi J Nasir A Coppola D Chen D-T. First-line chemotherapy with capecitabine and temozolomide in patients with metastatic pancreatic endocrine carcinomas. Cancer. (2011) 117:268–75. 10.1002/cncr.25425 20824724 PMC4665634

[14] Byun J Kim SH Jeong HS Rhee Y Lee WJ Kang CM. ACTH-producing neuroendocrine tumor of the pancreas: a case report and literature review. Ann Hepatobiliary Pancreat Surg. (2017) 21:61–65. 10.14701/ahbps.2017.21.1.61. 28317048 PMC5353908

[15] Kamp K Alwani RA Korpershoek E Franssen GJ De Herder WW Feelders RA. Prevalence and clinical features of the ectopic ACTH syndrome in patients with gastroenteropancreatic and thoracic neuroendocrine tumors. Eur J Endocrinol. (2016) 174:271–80. 10.1530/EJE-15-0968 26643855

